# Comparison of the effects of sibling and parental history of type 2 diabetes on metabolic syndrome

**DOI:** 10.1038/s41598-020-79382-z

**Published:** 2020-12-17

**Authors:** Hsuan Chiu, Mei-Yueh Lee, Pei-Yu Wu, Jiun-Chi Huang, Szu-Chia Chen, Jer-Ming Chang

**Affiliations:** 1 Department of General Medicine, Kaohsiung Medical University Hospital, Kaohsiung, Taiwan; 2 Division of Endocrinology and Metabolism, Department of Internal Medicine, Kaohsiung Medical University Hospital, Kaohsiung Medical University, Kaohsiung, Taiwan; 3 Division of Nephrology, Department of Internal Medicine, Kaohsiung Medical University Hospital, Kaohsiung Medical University, Kaohsiung, Taiwan; 4 Department of Internal Medicine, Kaohsiung Municipal Siaogang Hospital, Kaohsiung Medical University, 482, Shan-Ming Rd., Hsiao-Kang Dist., Kaohsiung, 812 Taiwan, ROC; 5 Faculty of Medicine, College of Medicine, Kaohsiung Medical University, Kaohsiung, Taiwan; 6 Research Center for Environmental Medicine, Kaohsiung Medical University, Kaohsiung, Taiwan

**Keywords:** Endocrinology, Health care

## Abstract

The aim of this study was to investigate the associations between sibling history, parental history and simultaneous sibling and parental history of diabetes, and the presence of the metabolic syndrome (MetS) and its components. Our study comprised 5000 participants from Taiwan Biobank until April, 2014. The participants were stratified into four groups according to sibling and/or parental family history (FH) of DM. MetS was defined as having 3 of the following 5 abnormalities based on the standard of the NCEP ATP III and modified criteria for Asians. The prevalence of MetS and its traits was estimated and compared among the four familial risk strata. Multivariate logistic regression analysis showed participants with sibling FH of DM [vs. no FH of DM; odds ratio (OR) 1.815; 95% confidence interval (CI) 1.293 to 2.548; *p* = 0.001], participants with parental FH of DM (vs. no FH of DM; OR 1.771; 95% CI 1.468 to 2.135; *p* < 0.001), and participants with simultaneous sibling and parental FH of DM (vs. no FH of DM; OR 2.961; 95% CI 2.108 to 4.161; *p* < 0.001) were significantly associated with MetS. A synergistic effect of sibling FH of DM and parental FH of DM on the association of MetS was also observed. In a nationally representative sample of Taiwan adults, a simultaneous sibling and parental history of diabetes shows a significant, independent association with MetS and its components, except for abdominal obesity. The association highlights the importance of obtaining stratified FH information in clinical practice and may help to identify individuals who should be targeted for screening and early prevention of MetS.

## Introduction

The ever increasing prevalence of diabetes mellitus (DM) and its complications continues to increase the burden on healthcare systems worldwide^1^. DM develops due to interactions between hereditary and environmental exposure^2^. A family history (FH) is an useful screening tool that can provide both valuable genetic information and also common environmental component factors^3,4^. Previous studies have reported a significant association between a positive FH of DM and an increased risk of type 2 DM^5–7^. In addition, several studies have shown that individuals with a FH of type 2 DM have high triglyceride and low-density lipoprotein (LDL) cholesterol levels, low high-density lipoprotein (HDL) cholesterol levels, high rates of general and central obesity, high anthropometric values [waist-to-hip ratio and body mass index (BMI)], insulin resistance, and an increased risk of hypertension compared to individuals without a FH of type 2 DM^8–10^. The combination of obesity (and particularly central obesity), glucose intolerance, dyslipidemia and hypertension is termed the “metabolic syndrome (MetS).” These findings support the hypothesis that shared genes and common environmental exposure contribute to complex disorders such as DM and MetS. Some clinical investigations have also revealed an association between a positive FH of diabetes and the prevalence of MetS^11–13^.

FH includes both parental FH and sibling FH, and different categories of FH have been shown to have different impacts on certain subclinical diseases^14,15^. The profile of FH has also been used in several studies to investigate the association between a FH of diabetes and the risk of the disease^16,17^. Another study conducted in Taiwan concluded that a sibling history of DM was more important than a parental history when assessing the risk of diabetes^18^. However, no studies have compared the effect of sibling FH, parental FH and the combination of sibling FH and parental FH on MetS and MetS components. Therefore, the aims of the present study were to investigate the association between a stratified FH of DM, including a sibling FH, parental FH and both sibling and parental FH of DM, and the presence of MetS in 5000 participants from the Taiwan Biobank (TWB) study.

## Results

The mean age of the 5000 participants (2335 males and 2665 females) was 49.6 ± 10.7 years. The overall prevalence rate of MetS was 19.1%. The participants were stratified into four groups according to a sibling and/or parental FH of DM as follows: and/or parental FH of DM as follows: no FH (n = 3279), sibling FH only (n = 242), parental FH only (n = 1266), and both sibling and parental FH (n = 213). A comparison of the clinical characteristics among these study groups is shown in Table 1. Compared to the participants with no FH of DM, those with both a sibling and parental FH of DM were older and had a lower prevalence of smoking history, higher prevalence of DM, higher prevalence of hypertension, shorter height, higher WC, higher waist-to-hip ratio, higher SBP, higher fasting glucose level, higher triglyceride level, and higher prevalence of regular exercise habits. In addition, the participants with both a sibling and parental FH of DM had a higher prevalence rate of MetS and of each MetS component except for a low level of HDL-cholesterol.

**Table 1:** Comparison of clinical characteristics among study groups according to sibling and/or parental FH of DM.

Characteristics	No FH of DM (n = 3279)	Sibling FH of DM (n = 242)	Parental FH of DM (n = 1266)	Simultaneous sibling and parental FH of DM (n = 213)
Age (year)	49.1 ± 10.9	55.6 ± 8.5*	48.5 ± 10.0^†^	55.9 ± 8.0*^#^
Male gender (%)	48.2	38.4*	45.3	40.8
Smoking history (%)	28.7	27.3	26.1	20.2*
DM (%)	2.9	14.0*	5.9*^†^	22.5*^†#^
Hypertension (%)	10.4	16.9*	10.0^†^	25.8*^†#^
Coronary artery disease (%)	1.0	0.4	1.5	0.9
Cerebrovascular disease (%)	0.4	1.7	0.6	0.5
Height (cm)	162.8 ± 8.4	161.0 ± 7.7*	162.6 ± 8.4^†^	160.9 ± 8.0*^#^
Weight (kg)	64.1 ± 12.1	63.7 ± 11.3	65.2 ± 12.6*	63.2 ± 10.3
BMI (kg/m^2^)	24.1 ± 3.5	24.5 ± 3.2	24.5 ± 3.6*	24.3 ± 3.0
WC (cm)	83.8 ± 9.7	85.6 ± 9.2*	84.5 ± 9.6	85.8 ± 8.4*
HC (cm)	96.3 ± 6.5	96.2 ± 6.4	96.9 ± 6.8*	96.4 ± 6.4
Waist-to-hip ratio	0.87 ± 0.07	0.89 ± 0.06*	0.87 ± 0.06^†^	0.89 ± 0.06*^#^
SBP (mmHg)	114.9 ± 17.0	120.6 ± 16.3*	114.8 ± 17.0^†^	121.3 ± 18.6*^#^
DBP (mmHg)	71.3 ± 11.1	72.0 ± 10.1	71.4 ± 11.4	72.8 ± 10.6
**Laboratory parameters**				
Fasting glucose (mg/dL)	94.8 ± 16.7	104.0 ± 31.5*	98.2 ± 20.9*^†^	107.6 ± 30.3*^#^
Triglyceride (mg/dL)	95 (67–136)	101 (73.75–139.25)	99.5 (69.75–146)*	110 (81.5–163.5)*^#^
Total cholesterol (mg/dL)	194.9 ± 36.0	200.5 ± 36.0	194.0 ± 35.7	196.8 ± 37.2
HDL-cholesterol (mg/dL)	54.7 ± 13.4	54.12.80 ± 13.9	53.3 ± 13.2*	53.2 ± 12.8
LDL-cholesterol (mg/dL)	122.1 ± 32.5	125.6 ± 32.3	121.6 ± 31.9	121.7 ± 32.6
Hemoglobin (g/dL)	1 1.44.0 ± 1.6	14.0 ± 1.5	14.0 ± 1.6	13.9 ± 1.4
eGFR (mL/min/1.73 m^2^)	107.2 ± 24.5	107.4 ± 25.9	109.2 ± 26.2	108.7 ± 25.0
Uric acid (mg/dL)	5.6 ± 1.5	5.6 ± 1.5	5.6 ± 1.5	5.6 ± 1.3
MetS (%)	15.7	28.5*	23.5*	35.2*^#^
**MetS component**				
Abdominal obesity (%)	44.1	53.7*	49.0*^†^	54.0*^†#^
Hypertriglyceridemia (%)	20.0	21.5	23.9*	29.1*
Low HDL-cholesterol (%)	21.3	25.2	26.1*	29.1
Hyperglycemia (%)	19.5	37.6*	28.4*^†^	50.7*^†#^
High blood pressure (%)	22.2	35.1*	23.8*^†^	39.0*^†#^
Regular exercise habits (%)	43.5	56.6*	43.4^†^	55.4*^#^
Midnight snack habits (%)	30.8	25.2	31.2	29.6

*FH* family history, *DM* diabetes mellitus, *BMI* body mass index, *WC* waist circumference, *HC* hip circumference, *SBP* systolic blood pressure, *DBP* diastolic blood pressure, *HDL* high-density lipoprotein, *LDL* low-density lipoprotein, *eGFR* estimated glomerular filtration rate, *MetS* metabolic syndrome.

*p < 0.05 compared with no FH of DM.

^†^*p* < 0.05 compared with sibling FH of DM.

^#^*p* < 0.05 compared with parental FH of DM.

### Determinants of MetS

**Figure 1:**
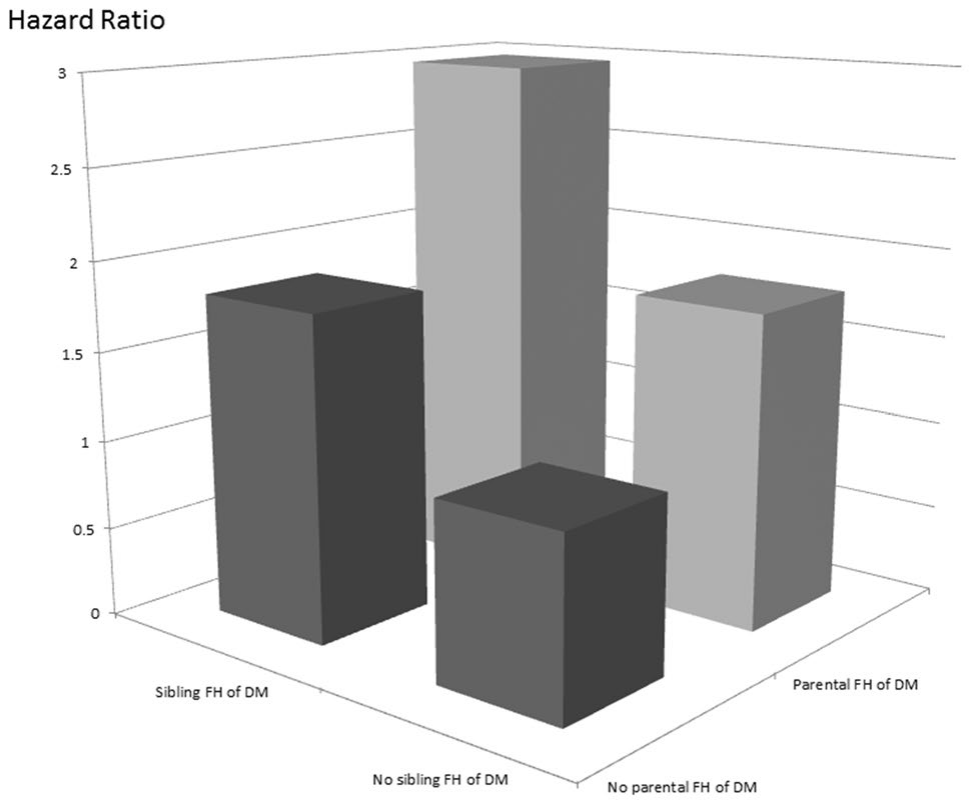
Synergistic effect of sibling FH and parental FH of DM on the association of MetS.

Table 2 shows the determinants of MetS in the study participants. After adjusting for the FH of DM groups, age, sex, smoking history, BMI, hemoglobin, eGFR, uric acid, regular exercise and midnight snacking habits, the participants with a sibling FH of DM [vs. no FH of DM; odds ratio (OR) 1.815; 95% confidence interval (CI) 1.293 to 2.548; *p* = 0.001], a parental FH of DM (vs. no FH of DM; OR 1.771; 95% CI 1.468 to 2.135; *p* < 0.001), and both a sibling and parental FH of DM (vs. no FH of DM; OR 2.961; 95% CI 2.108 to 4.161; *p* < 0.001) were significantly associated with MetS. In addition, old age, female sex, high BMI, high hemoglobin, high eGFR, high uric acid, and midnight snacking habits were independently associated with MetS. A synergistic effect of a sibling FH of DM and parental FH of DM on the association with MetS was observed (Fig. 1).

**Table 2:** Determinants of MetS using multivariable logistic regression analysis.

Parameter	Multivariable	
	Odds ratio (95% CI)	*p*
**FH of DM**		
No FH of DM	Reference	
Sibling FH of DM	1.815 (1.293–2.548)	0.001
Parental FH of DM	1.771 (1.468–2.135)	< 0.001
Simultaneous sibling and parental FH of DM	2.961 (2.108–4.161)	< 0.001
Age (per 1 year)	1.061 (1.052–1.071)	< 0.001
Male (*vs.* female)	0.463 (0.359–0.597)	< 0.001
Smoking history	1.177 (0.956–1.448)	0.125
BMI (per 1 kg/m^2^)	1.388 (1.349–1.428)	< 0.001
Hemoglobin (per 1 g/dL)	1.162 (1.081–1.250)	< 0.001
eGFR (per 1 mL/min/1.73 m^2^)	1.005 (1.002–1.009)	0.005
Uric acid (per 1 mg/dL)	1.321 (1.233–1.415)	< 0.001
Regular exercise habits (%)	0.935 (0.785–1.113)	0.447
Midnight snack habits (%)	1.216 (1.014–1.457)	0.035

Values expressed as odds ratio and 95% confidence interval (CI). Abbreviations are the same as in Table 1.

Adjusted for study groups of FH of DM, age, sex, smoking history, BMI, hemoglobin, eGFR, uric acid, regular exercise and midnight snack habits.

### Determinants of each component of MetS

Table 3 shows the determinants of each component of MetS in the study participants, as below:

1. Abdominal obesity (WC > 90 cm for men and > 80 cm for women)
  Compared to the participants with no FH of DM, those with a sibling FH of DM, a parental FH of DM, and both a sibling and parental FH of DM were all associated with abdominal obesity in unadjusted analysis. However, the associations were no longer significant after multiple adjustments.
2. Hypertriglyceridemia (triglyceride level ≥ 150 mg/dL)
  After multiple adjustments, compared to the participants with no FH of DM, those with a parental FH of DM (OR 1.252; 95% CI 1.058 to 1.481; *p* = 0.009) and both a sibling and parental FH of DM (OR 1.856; 95% CI 1.333 to 2.586; *p* < 0.001) were independently associated with hypertriglyceridemia. However, the participants with a sibling FH of DM were not.
3. A low level of HDL-cholesterol (< 50 mg/dL in women and < 40 mg/dL in men)
  After multiple adjustments, compared to the participants with no FH of DM, those with a parental FH of DM (OR 1.207; 95% CI 1.030 to 1.414; *p* = 0.020) and both a sibling and parental FH of DM (OR 1.415; 95% CI 1.021 to 1.962; *p* = 0.037) were independently associated with a low level of HDL-cholesterol. However, the participants with a sibling FH of DM were not.
4. Hyperglycemia (fasting whole-blood glucose concentration ≥ 110 mg/dL or a diagnosis of DM)
  After multiple adjustments, compared to the participants with no FH of DM, those with a sibling FH of DM (OR 2.069; 95% CI 1.543 to 2.774; *p* < 0.001), a parental FH of DM (OR 1.777; 95% CI 1.510 to 2.090; *p* < 0.001), and both a sibling and parental FH of DM (OR 3.765; 95% CI 2.785 to 5.090; *p* < 0.001) were all independently associated with hyperglycemia.
5. High blood pressure (SBP ≥ 130 mmHg, DBP ≥ 85 mmHg, or a diagnosis or treatment for hypertension)
  After multiple adjustments, compared to the participants with no FH of DM, those with a sibling FH of DM (OR 1.459; 95% CI 1.080 to 1.972; *p* = 0.014) and both a sibling and parental FH of DM (OR 1.774; 95% CI 1.299 to 2.423; *p* < 0.001) were associated with high blood pressure. However, the participants with a parental FH of DM were not.

**Table 3:** Relation of study groups to each MetS components using multivariable logistic regression analysis.

Study groups	Abdominal obesity		Hypertriglyceridemia		Low HDL-cholesterol		Hyperglycemia		High blood pressure	
	Odds ratio (95% CI)	*p*	Odds ratio (95% CI)	*p*	Odds ratio (95% CI)	*p*	Odds ratio (95% CI)	*p*	Odds ratio (95% CI)	*p*
**Unadjusted**										
No FH of DM	Reference		Reference		Reference		Reference		Reference	
Sibling FH of DM	1.471 (1.132–1.912)	0.004	1.094 (0.796–1.505)	0.579	1.242 (0.918–1.679)	0.160	2.485 (1.889–3.269)	< 0.001	1.897 (1.439–2.501)	< 0.001
Parental FH of DM	1.217 (1.068–1.385)	0.003	1.258 (1.078–1.469)	0.004	1.304 (1.122–1.516)	0.001	1.632 (1.405–1.896)	< 0.001	1.093 (0.938–1.274)	0.256
Simultaneous sibling and parental FH of DM	1.488 (1.126–1.965)	0.005	1.642 (1.207–2.233)	0.002	1.513 (1.113–2.057)	0.008	4.241 (3.199–5.624)	< 0.001	2.237 (1.678–2.982)	< 0.001
**Multivariable adjusted**										
No FH of DM	Reference		Reference		Reference		Reference		Reference	
Sibling FH of DM	0.905 (0.624–1.312)	0.598	1.063 (0.752–1.502)	0.730	1.111 (0.807–1.531)	0.518	2.069 (1.543–2.774)	< 0.001	1.459 (1.080–1.972)	0.014
Parental FH of DM	1.015 (0.843–1.223)	0.874	1.252 (1.058–1.481)	0.009	1.207 (1.030–1.414)	0.020	1.777 (1.510–2.090)	< 0.001	1.146 (0.968–1.358)	0.114
Simultaneous sibling and parental FH of DM	1.085 (0.740–1.590)	0.677	1.856 (1.333–2.586)	< 0.001	1.415 (1.021–1.962)	0.037	3.765 (2.785–5.090)	< 0.001	1.774 (1.299–2.423)	< 0.001

Values expressed as odds ratio, hazard ratio and 95% confidence interval (CI). Abbreviations are the same as in Table 1.

Multivariable model: adjusted for study groups of FH of DM, age, sex, smoking history, BMI, hemoglobin, eGFR, uric acid, regular exercise and midnight snack habits.

**Figure 2:**
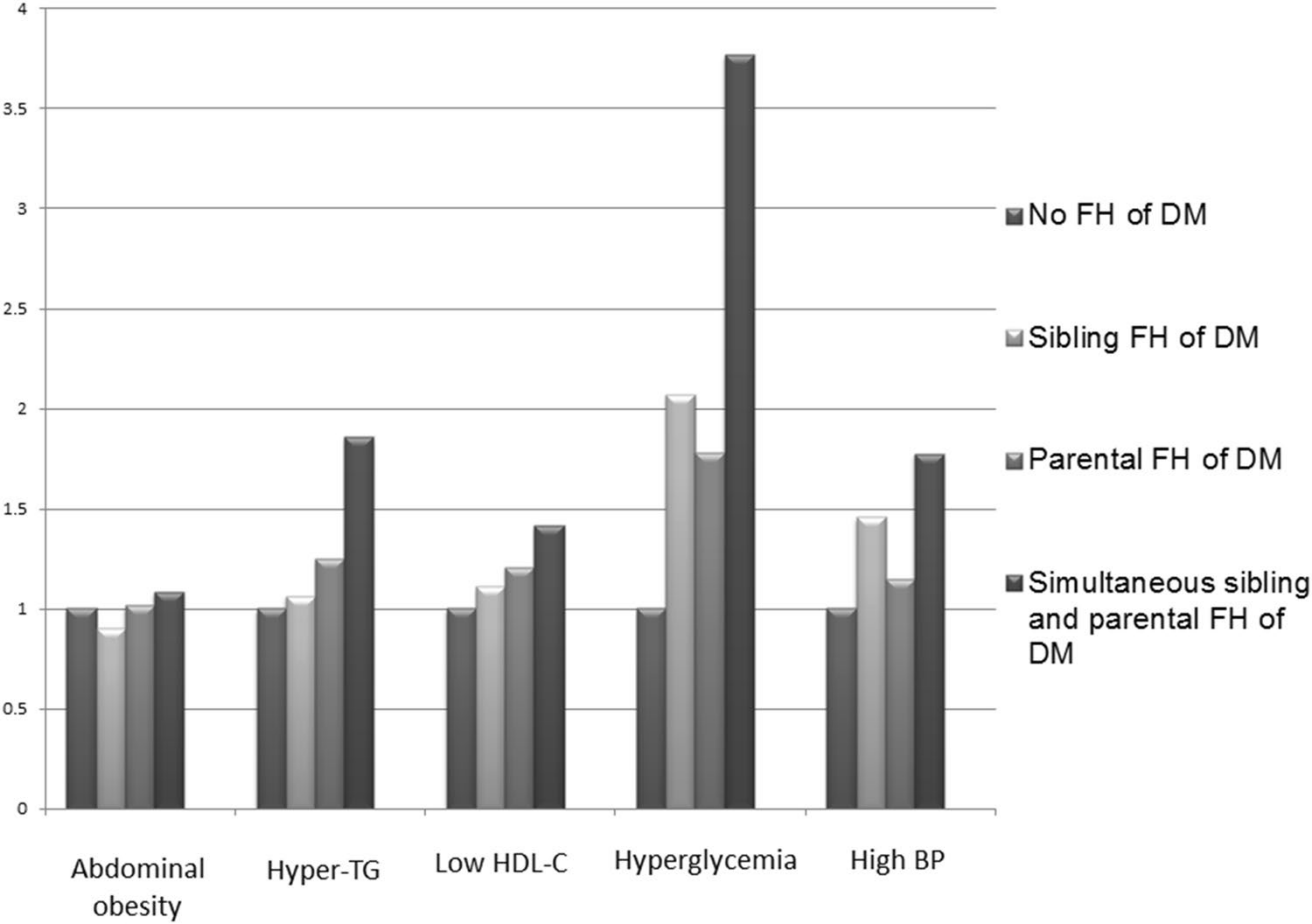
The correlation between the study groups with each component of MetS. Synergistic effect of sibling FH and parental FH of DM on the association of hyperglycemia component of MetS.

Figure 2 illustrates the correlations between the study groups with each component of MetS. A synergistic effect of a sibling FH of DM and parental FH of DM on the association with the hyperglycemia component of MetS was observed.

## Discussion

The present study of 5000 participants in the TWB showed that a FH of DM was significantly associated with MetS, regardless of whether there was a sibling or parental FH. In addition, individuals with both a sibling and parental FH of DM had the highest prevalence of MetS and the highest prevalence of each MetS component, except for abdominal obesity.

The first important finding of this study is that we observed a synergistic effect of a parental FH of DM and sibling FH of DM on the association with MetS and the component of hyperglycemia. In other words, the individuals whose parents and siblings had DM had a higher rate of MetS compared to those with no FH of DM. Several hypotheses could explain our findings. First, MetS is influenced by complex interactions between genetic and shared environmental factors. Patients and their relatives with type 2 DM often have metabolic abnormalities, including obesity, hypertension, hypertriglyceridemia and low HDL-cholesterol^8,19^. Several studies have also reported a significant association between a parental FH of DM and the prevalence of MetS. Rodriguez-Moran et al. reported that Mexican individuals with a parental FH of DM had a 3.5-fold higher risk of MetS than those with no FH of DM^20^. In addition, the Atherosclerosis Risk in Communities (ARIC) study showed that a positive parental FH of DM was associated with MetS and its components^21^. In a study of an Indian cohort conducted by Das et al., both a parental FH and either parental/sibling FH of DM were associated with an increased odds of MetS^13^. Moreover, individuals with a parental history of DM have also been reported to be more likely to develop MetS or insulin resistance^22^. Our results are consistent with previous reports that MetS is a heritable trait. Although the magnitude of the genetic effects of sibling and parent–child correlations may be similar, sibling-sibling correlations for most environmental risk factors of MetS are generally greater than parent–offspring correlations^23^. Sibling pairs have been shown to share more environmental factors than between parents and their offspring, and several common environmental factors during young childhood have been associated with the subsequent development of diseases in adulthood^24^. In addition, previous studies have also demonstrated an association between familial aggregation and a higher risk of develop MetS in siblings^23,25–27^. Second, insulin resistance has been proposed to be the underlying cause of MetS and its components^28^. Insulin resistance has been proposed to cause hyperglycemia by decreasing glucose uptake in muscles, and also dyslipidemia (i.e. a high concentration of triglycerides and low concentration of HDL-cholesterol) caused by an increase in transport of free fatty acids to the liver due to an increase in adipocyte lipolysis^29^. In addition, insulin resistance has been proposed to be associated with hypertension, possibly due to vascular changes, increased salt sensitivity, and endothelial dysfunction due to hyperinsulinemia^30^. Insulin resistance is also thought to play a key role in the pathogenesis of type 2 DM. Individuals with a FH of DM have been shown to be more likely to develop DM than those without a FH of DM^31–33^. Moreover, a FH of DM has been reported to have a graded association with DM, because the prevalence of diabetes increases with the number of generations with a FH^34^. Taken together, our observation of the synergistic effect of FH of DM on the association with MetS indicates the impacts of shared genetic and environmental factors as well as familial aggregation on MetS.

Another important finding of the present study is that a sibling FH of DM was more strongly correlated with the MetS component of high blood pressure than a parental FH of DM. Katulanda et al. reported that a FH of DM was associated with the prevalence of hypertension in a cohort of Sri Lankan adults^34^. In addition, Pontiroli et al. demonstrated that higher SBP and DBP were common in siblings of parents with DM^35^. In addition, individuals with a higher number of generations affected by diabetes also had higher SBP and DBP. On the other hand, Shirakawa et al. reported that a FH of DM did not increase the risk of hypertension in male Japanese workers^36^. Another study of Taiwanese patients reported that a parental FH of DM was associated with a lower risk of hypertension in patients with type 2 DM^37^. It is not clear why a sibling FH of DM was more strongly associated with hypertension than a parental history. The progression of hypertension is influenced by both genetic factors and common familial behavior and lifestyle factors such as smoking status, eating and drinking habits, and physical activity. A previous study suggested that an increased prevalence of hypertension among the relatives of diabetic patients may be caused by common genetic and/or environmental backgrounds^38^. However, another report suggested that the phenotypic correlations of blood pressure, and especially SBP, were mainly due to shared environmental factors^26^. As mentioned, sibling pairs have more shared environmental factors than parents and their offspring. Our findings may support that, in addition to genetic factors, environmental factors in a shared household among siblings may be equally important. Therefore, our observation of a higher odds ratio of hypertension for a sibling FH of DM than a parental FH suggests a greater sharing of environmental factors between siblings than between parents and their offspring, or that both dominant and additive genetic effects are involved.

The last important finding of this study is that a parental FH of DM was strongly associated with the MetS components of low HDL-cholesterol and hypertriglyceridemia, whereas a sibling FM of DM was not. A parental FH of DM was independently associated with both hypertriglyceridemia and low HDL-cholesterol, suggesting the possibility of a shared genetic etiology for these two traits. Previous studies have reported significant heritability for concentrations of HDL-cholesterol and triglycerides^39,40^. Individuals with a positive FH of DM have been reported to have significantly higher levels of triglycerides, and it is well known that insulin resistance also occurs in people without DM with endogenous hypertriglyceridemia^41^. Moreover, studies on first-degree relatives of patients with DM have also reported that those at an increased risk of DM have higher levels of serum triglycerides^42^. Moreover, in a study of 1113 probands in East Finland, first-degree relatives of patients with non-insulin-dependent diabetes had lower HDL-cholesterol levels and higher triglyceride levels compared to those of normoglycemic relatives without a FH of DM^43^. Our results suggest that, despite the established causal relationships between environmental factors and MetS components, genetic variation was still the dominant source of phenotypic variation in these components. However, our findings do not rule out the importance of lifestyle modification to lower the risk of MetS.

There are several limitations to this study. First, the cross-sectional design means that we cannot establish any temporal associations, and therefore the specific roles of a FH of DM in the development of MetS could not be determined with certainty. Follow-up studies are needed to confirm our results. Second, since the participants in this study were all of Chinese ethnicity, the findings may not be generalizable to other ethnicities. Third, since we did not assess the genetic information of the siblings and parents, it is difficult to estimate the relative impact of genetic factors on the prevalence of MetS. Finally, we only recorded physical activity intensity and midnight snacking habits with regards to lifestyle habits. Although, physical activity and dietary factors are important determinants for MetS, whether the participants and their families had the same lifestyle habits cannot be guaranteed.

In conclusion, the results of this study demonstrated strong and independent associations between a FM of DM and the prevalence of MetS in a Taiwanese population. Moreover, both a sibling and parental FH of DM was more relevant than either a sibling FH of DM or parental FH of DM alone. This association highlights the importance of obtaining FH information in clinical practice and the potential of FH as a useful approach for the assessment of the risk of MetS and to aid in its prevention.

## Materials and methods

### Ethics statement

The TWB received ethical approval from the Institutional Review Board on Biomedical Science Research/IRB-BM, Academia Sinica, Taiwan, and from the Ethics and Governance Council of TWB, Taiwan. Written informed consent was obtained from each participant in accordance with institutional requirements and the principles of the Declaration of Helsinki. Moreover, the current study was approved by the Institutional Review Board of Kaohsiung Medical University Hospital (KMUHIRB-E(I)-20180242).

### TWB

The TWB was established with the aim of collecting lifestyle and genomic data from residents of Taiwan, and it is currently the largest biobank supported by the government in Taiwan^44,45^. The TWB includes data on community-based volunteers aged 30–70 years with no history of cancer. In this study, we included 5000 individuals from the TWB up to April 2014, all of whom provided written informed consent, blood samples, and other information via face-to-face interviews and physical examinations.

The TWB includes data on body height, weight, waist circumference (WC) and hip circumference (HC). BMI was calculated as weight (kg)/height (m)^2^, and the waist-to-hip ratio was also calculated. The face-to-face interview with one of the TWB researchers included a questionnaire which asked about personal information, lifestyle factors, diet and personal and family medical history. The participants were also asked about exercise habits, and regular exercise was defined as performing a physical activity for at least 30 min. In this study, only leisure activities such as swimming, playing basketball, jogging, hiking, cycling, yoga, and exercise-based computer games were defined as “exercise”, and occupational activities were not included.

### Collection of demographic, medical and laboratory data

The following variables were recorded at baseline: demographic features (age and sex), smoking history, medical history (cerebrovascular diseases, DM, coronary artery disease and hypertension), examination findings [systolic (SBP) and diastolic blood pressures (DBP)], and laboratory data [fasting glucose, triglycerides, total cholesterol, HDL-cholesterol, LDL-cholesterol, hemoglobin, estimated glomerular filtration rate (eGFR) and uric acid]. EGFR was calculated using the Modification of Diet in Renal Disease 4-variable equation^46^.

### Definition of MetS

MetS was defined as the presence of three of the following five abnormalities according to the NCEP-ATP III^47^ and modified criteria for Asians^48^: (1) abdominal obesity (defined as WC > 80 cm in women and > 90 cm in men); (2) hypertriglyceridemia (defined as a triglyceride level ≥ 150 mg/dL); (3) low concentration of HDL-cholesterol (defined as < 50 mg/dL in women and < 40 mg/dL in men); (4) hyperglycemia (defined as fasting whole-blood glucose concentration ≥ 110 mg/dL or a diagnosis of DM); and (5) high blood pressure (defined as DBP ≥ 85 mmHg, SBP ≥ 130 mmHg, or the presence of hypertension diagnosed or treated by a physician).

### Statistical analysis

Statistical analysis was performed using SPSS version 19.0 for Windows (SPSS Inc. Chicago, USA). Data were expressed as percentage, mean ± standard deviation, or median (25th–75th percentile) for triglycerides. One-way analysis of variance followed by Bonferroni's post hoc test was used for multiple comparisons among groups. The study participants were stratified into four groups according to a sibling and/or parental FH of DM. Multivariate logistic regression analysis was used to identify associations between the participants with MetS and each MetS component. A *p* value of less than 0.05 was considered to indicate a statistically significant difference.
